# Association Between a Wider Availability of Health Information and Health Care Utilization in Vietnam: Cross-Sectional Study

**DOI:** 10.2196/jmir.8328

**Published:** 2017-12-18

**Authors:** Hoang Thuy Linh Nguyen, Keiko Nakamura, Kaoruko Seino, Van Thang Vo

**Affiliations:** ^1^ Department of Global Health Entrepreneurship Tokyo Medical and Dental University Tokyo Japan; ^2^ Faculty of Public Health Hue University of Medicine and Pharmacy Hue Viet Nam; ^3^ Institute for Community Health Research Hue University of Medicine and Pharmacy Hue Viet Nam

**Keywords:** health care utilization, health information, mass media, Internet

## Abstract

**Background:**

The rapid and widespread development of mass media sources including the Internet is occurring worldwide. Users are being confronted with a flood of health information through a wide availability of sources. Studies on how the availability of health information has triggered users’ interest in utilizing health care services remain limited within the Vietnamese population.

**Objective:**

This study examined the associations between the wider availability of sources for health information and health care utilization in Vietnam after adjusting for potential confounding variables.

**Methods:**

The data for this study were drawn from a cross-sectional study conducted over a 6-month period in Hue, a city in central Vietnam. The participants were 993 randomly selected adults aged between 18 and 60 years. Information was collected through face-to-face interviews on the types of information sources that were consulted, including traditional media (television), Internet, and health education courses, as well as the impact of such information on health care use (emergency department visits, hospitalizations, doctor visits). Multivariable logistic regression analyses were performed at a 95% confidence level.

**Results:**

The prevalence of watching television, using the Internet, and attending health education courses to obtain health information were 50.9% (505/993), 32.9% (327/993), and 8.7% (86/993), respectively. After further adjustments for self-reported health status, the presence of health insurance, and monthly income, respondents who watched television and used the Internet to obtain health information were 1.7 times more likely to visit a doctor (television: adjusted odds ratio [AOR] 1.69, 95% CI 1.30-2.19; Internet: AOR 1.64, 95% CI 1.23-2.19), and also significantly associated with inpatient hospitalization (*P*=.003).

**Conclusions:**

The use of widely available mass media sources (eg, television and the Internet) to obtain health information was associated with higher health care utilization. How this interest in health-related information can be used so that it will have a beneficial effect on care-seeking behavior should be a topic of concern to further health promotion in developing countries.

## Introduction

Searching for health information is becoming more common and people no longer passively trust the advice of specialized physicians. The rapid development of digital media has facilitated an increase in the amount of health information that the public can easily access. Traditional media sources, such as television, radio, and newspapers, have developed their medical sections to adapt to the public’s concern over health issues [1]. In addition, there is a plethora of information available online and searching the Internet for health purposes has become common among the general public in recent years. Approximately 60% of total Internet users in the US Health Information National Trends Survey reported using the Internet to search for health information [2]. Thus, compared with obtaining health information directly from a doctor, traditional media and Internet, which were generally identified as mass media sources, can provide diverse health information and allow the general population to confirm what they have heard, thereby contributing to improved decisions regarding their health [3,4].

Previous studies have examined the patterns of health care use within the Behavioral Model of Health Care Utilization [5]. The potential effect of seeking health information through mass media on the public’s use of health care is a focus of health policy makers. Understanding the relationship between the use of health information sources and health care utilization is necessary because well-informed patients have a better ability to cope with diseases and to make good decisions. A relevant study has suggested that the likelihood that an individual would visit a physician increases by approximately 10% for every 1-hour increment spent searching the Internet for medical information [6]. However, other studies have reported a negative or lack of effect between the use of mass media to obtain medical information and the number of outpatient clinic visits. Based on a survey in Japan, nearly 90% of the respondents thought that their Internet use was not associated with the frequency at which they visited outpatient clinics or made phone calls to their doctors to inquire about health-related issues [7]. Thus, the effect of using mass media sources to obtain health-related information on the actual use of health services remains debatable.

Vietnam is a developing country in Southeast Asia that has successfully placed the growth of information and communication technology industries near the forefront of its economic priorities over the past 15 years. Broadcast media in Vietnam has been undergoing modernization on a daily basis. In parallel, the number of Internet users has rapidly increased from approximately 33 million in 2013 to 44.4 million in 2015 and is presently estimated to reach 65.7 million in 2021 [8-10]. These users are being confronted with a burst of health-related information through a wider availability of sources. Social programs on television and the Internet emphasize not only socioeconomic policy-related news and entertainment, but also health education programs to adapt to the public’s increasing interest in seeking health information and using health services. Vietnam’s government-run TV stations (national and provincial) always have health education programs at least once per week and sometimes give updates on health news, such as infectious disease epidemics, treatment, etc, in the daily news. However, studies on how the surging availability of information related to medicine affects the decisions of users to utilize health care services remain limited for the Vietnamese population. Thus, the aim of this study was to examine the association between the wider availability of health information sources and health care utilization after controlling for potential confounding variables.

## Methods

### Study Area

The study was conducted in the city of Hue, which is in the central area of Thua Thien-Hue Province in the central region of Vietnam. The city of Hue has 27 administrative quarters, and its population was estimated to be approximately 354,556 in 2015.

### Study Design

We conducted a cross-sectional survey from May to September 2015, using a structured questionnaire that was administered to adults aged between 18 and 60 years.

The participants were selected based on the following criteria:

1. A multistage, stratified cluster random sampling design was used. First, six quarters were randomly selected from among the 27 quarters of the city of Hue (three quarters were selected from urban areas, and three quarters were selected from suburban areas). A total of 30 subgroups were randomly selected from 112 small administrative units of six quarters in the survey (ie, five subgroups per quarter). Second, approximately 18 to 60 adults in each subgroup were systematically selected from a booklet listing the households.
2. The inclusion criteria were age between 18 and 60 years, a history of living in the city of Hue for more than 6 months, the ability to communicate or read, and the ability to meet with the investigators.
3. The exclusion criteria were the presence of mental disorders, cognitive disorders, or an unwillingness to participate.

A total of 1005 participants agreed to participate and the valid response rate was 98.52% (1005/1020). The 12 senior medical students recruited had 1 day of thorough training before visiting selected households. They visited households on weekends and did face-to-face interviews of the individuals. Finally, 993 participants were included in the analysis (12 participants with missing values were excluded).

### Measurement

This survey contained a set of questions about participant’s characteristics, the availability of health information sources, health care utilization, and others. The questionnaire was developed from the Health Literacy Survey-Asia-Questionnaire (HLS-Asia-Q), which followed the methodology of the full version of the European HLS Questionnaire (HLS-EU-Q) [11] and has been validated in Asia including Vietnam [12]. This questionnaire included minor questions vetted by public health experts so that it would fit the local context, and then it was pretested for readability and understandability by experienced survey researchers before use in the field.

Demographic and socioeconomic characteristics were identified via the questionnaire, including age (years), sex (male, female), marital status (never married vs married, divorced, or widowed), highest education attainment in the formal education system (junior high school and below, senior high school, or university and above), monthly income in local currency Vietnam dong (1 million VND=US $44.50; <3 million VND, 3-7 million VND, or >7 million VND), and the presence of health insurance (yes/no). Heath-related characteristics were measured via self-reporting. The participants were asked to rate their overall health status using a five-point Likert scale ranging from 1=very bad to 5=very good. The self-reported health statuses were recorded as “poor,” “moderate,” or “good.”

The availability of health information sources, including mass media sources (watching television, Internet use) and health education courses, was measured via single self-reported items and separate questions (eg, “How frequently do you...?”) with possible responses of “often,” “sometimes,” “rarely,” and “never.” We defined “watching television to obtain health information” as watching medical-related TV series and using the Internet to obtain health information through a computer or mobile phone to search for medical information or update the health news on a Web browser or social networking service. The frequencies were dichotomized as “yes,” including often/sometimes, and “no,” including rarely/never (reference).

Health care utilization was defined as ambulatory visits to health care facilities including emergency department visits, hospitalizations, and doctor visits based on the responses to separate questions such as “How many times have you visited...in the last 12 months?” with possible responses of “0,” “1-2 times,” “3-5 times,” and “6 times or more.” These variables were recorded and classified as either none or ≥1 time.

### Statistical Analysis

The percentage of participants using mass media sources (television, Internet) and the attendance of health education courses to obtain health information were calculated.

During the descriptive analysis, categorical variables were summarized using proportions and then presented in tables. A bivariate analysis was performed to test for associations between the dependent variable “health care utilization” and other independent variables.

Multivariable logistic regression analysis by the enter method was done to evaluate the independent association between the outcome variables (emergency department visits, hospitalizations, doctor visits) and the availability of health information sources. For each outcome variable of health care utilization, we separately entered the independent variables (eg, watching television, Internet use, and attending health education) after adjustments for the possible influence of the self-reported health status, monthly income, and the presence of health insurance status in the logistic regression model. The Hosmer-Lemeshow goodness-of-fit test with *P*>.05 was used to assess the goodness-of-fit model. Odds ratios (ORs) assessed the strength of the associations; 95% confidence intervals and a *P* value of less than .05 were used for significance testing.

The data were identified and analyzed using SPSS version 23.0.

### Ethical Considerations

This study was approved by the Institutional Review Board of Hue University of Medicine and Pharmacy. The purpose of the study was explained on the first page of the questionnaire. All the enrolled participants agreed to cooperate with the investigators after the purpose of the research was explained, and responses were regarded as informed consent.

## Results

### Participant Characteristics

The characteristics of the 993 survey participants included in our analysis are summarized in Table 1. The mean age was 39.7 (SD 12.7) years, 57.9% (575/993) were female, most were married (75.3%,748/993), and 49.2% (504/993) had less than a junior high school education. In terms of wages, 51.4% (843/993) of respondents reported earning less than 3 million VND per month. A “poor” health status was reported by 8.0% (79/993) of the respondents; 84.9% (510/993) of participants had health insurance.

### Frequency of Wider Availability of Health Information Sources

Table 1 also shows the frequency of the availability of health-related information from various sources. Regarding watching medical-related TV programs, 43.4% (431/993) of the participants reported that they sometimes used such viewing for health purposes, and 7.5% (74/993) of them reported that they often used it for health purposes. The frequency of Internet use for obtaining health information was reported as sometimes by 26.2% (260/993) of the respondents and as often by 6.7% (67/993). Only 8.7% (86/993) of participants reported that they sometimes or often attended health education courses to obtain health information.

### Health Care Utilization and a Wider Availability of Information Sources for Health Purposes

In a bivariate analysis, we found that self-reported health status, the presence of health insurance, and monthly income were associated with emergency department visits, hospitalizations, and doctor visits (see Table 2). Self-reported health status was significantly associated with visiting health facilities including emergency department visits (*P*<.001), inpatient hospitalizations (*P*=.02), and doctor visits (*P*=.001). Those having health insurance had higher likelihoods of hospitalization (OR 1.93, 95% CI 1.16-3.20) and doctor visits (OR 3.15, 95% CI 2.16-4.60). Respondents with a monthly income of greater than 7 million VND were 2.4 times more likely to visit a doctor compared to those who had a monthly income of less than 3 million VND (OR 2.44, 95% CI 1.42-4.17).

Table 2 also shows an association between the availability of health information sources and health care visits. Obtaining health information by watching television or by using the Internet were significantly associated with inpatient hospitalization (*P*<.001 and *P*=.007, respectively) and doctor visits (*P*<.001). There was also a significant association between attending health education courses and inpatient hospitalization doctor visits (*P=*.002 and *P*=.02).

**Table 1:** Participant characteristics by health care utilization (N=993).

Participant characteristics		Total, n (%)	Emergency visit, n (%)	Hospitalization, n (%)	Doctor visit, n (%)
**Age (years)**					
	18-24	171 (17.2)	8 (13.6)	33 (16.3)	86 (16.7)
	25-34	195 (19.6)	13 (22.0)	47 (23.2)	101 (19.6)
	35-44	221 (22.3)	13 (22.0)	34 (16.7)	98 (19.1)
	45-54	243 (24.5)	15 (25.4)	46 (22.6)	134 (26.1)
	55-60	163 (16.4)	10 (17.0)	43 (21.2)	95 (18.5)
**Gender**					
	Male	418 (42.1)	22 (37.3)	81 (39.9)	189 (36.8)
	Female	575 (57.9)	37 (62.7)	122 (60.1)	325 (63.2)
**Legal marital status**					
	Unmarried	232 (23.4)	16 (27.1)	43 (21.2)	110 (21.4)
	Married	748 (75.3)	43 (72.9)	157 (77.3)	401 (78.0)
	Separated/divorced/widowed	13 (1.3)	0 (0.0)	3 (1.5)	3 (0.6)
**Education**					
	Below junior high school	489 (49.2)	32 (54.2)	86 (42.4)	220 (42.8)
	Senior high school	262 (26.4)	17 (28.8)	56 (27.6)	148 (28.8)
	Above senior high school	242 (24.4)	10 (17.0)	61 (30.0)	146 (28.4)
**Monthly income (VND)**^a^					
	<3 million	510 (51.4)	37 (62.7)	105 (51.7)	252 (49.0)
	3-7 million	412 (41.5)	20 (33.9)	80 (39.4)	212 (41.3)
	>7 million	71 (7.1)	2 (3.4)	18 (8.9)	50 (9.7)
**Self-reported health status**					
	Good	298 (30.0)	9 (15.3)	56 (27.6)	142 (27.6)
	Moderate	616 (62.0)	37 (62.7)	122 (60.1)	317 (61.7)
	Poor	79 (8.0)	13 (22.0)	25 (12.3)	55 (10.7)
**Health insurance**					
	Yes	843 (84.9)	51 (86.4)	184 (90.6)	471 (91.6)
	No	150 (15.1)	8 (13.6)	19 (9.4)	43 (8.4)
**Watching medical-related TV series**					
	Often	74(7.5)	4 (6.8)	33 (16.3)	52 (10.1)
	Sometimes	431(43.4)	28 (47.5)	93 (45.8)	250 (48.6)
	Rarely	295(29.7)	12 (20.3)	49 (24.1)	140 (27.3)
	Never	193(19.4)	15 (25.4)	28 (13.8)	72 (14.0)
**Getting medical-related information from the Internet**					
	Often	67 (6.7)	3 (5.1)	24 (11.8)	50 (9.7)
	Sometimes	260 (26.2)	15 (25.4)	59 (29.1)	147 (28.6)
	Rarely	189 (19.0)	8 (13.6)	33 (16.3)	96 (18.7)
	Never	477 (48.1)	33 (55.9)	87 (42.8)	221 (43.0)
**Attending health education courses**					
	Often	9 (0.9)	0 (0.0)	6 (3.0)	11 (2.1)
	Sometimes	77 (7.8)	3 (5.1)	23 (11.3)	41 (8.0)
	Rarely	115 (11.6)	4 (6.8)	18 (8.9)	52 (10.1)
	Never	792 (79.7)	52 (88.1)	156 (76.8)	410 (79.8)

^a^VND: Vietnamese Dong. 1 million VND=US $44.50.

**Table 2:** Association between health care utilization and respondents’ characteristics plus the availability of health information sources: unadjusted odds ratios.

Participants’ characteristics		Emergency visit		Hospitalization		Doctor visit		
		OR (95% CI)	*P*	OR (95% CI)	*P*		OR (95% CI)	*P*
**Self-reported health status**								
	Good	Ref		Ref			Ref	
	Moderate	2.05 (0.98-4.31)	.06	1.07 (0.75-1.52)	.72		1.17 (0.88-1.54)	.28
	Poor	6.33 (2.60-15.42)	<.001	2.00 (1.15-3.49)	.02		2.52 (1.48-4.28)	.001
**Health insurance**								
	No	Ref		Ref			Ref	
	Yes	1.14 (0.53-2.46)	.73	1.93 (1.16-3.20)	.01		3.15 (2.16-4.60)	<.001
**Monthly income (VND)**^a^								
	<3 million	Ref		Ref			Ref	
	3-7 million	0.65 (0.37-1.14)	.14	0.93 (0.67-1.29)	.66		1.09 (0.84-1.41)	.54
	>7 million	0.37 (0.09-1.57)	.18	1.31 (0.74-2.33)	.34		2.44 (1.42-4.18)	.001
**Watching medical-related TV series**								
	No	Ref		Ref			Ref	
	Yes	1.16 (0.68-1.96)	.59	1.78 (1.29-2.43)	<.001		1.94 (1.51-2.49)	<.001
**Getting medical-related information from the Internet**								
	No	Ref		Ref			Ref	
	Yes	0.89 (0.50-1.57)	.68	1.55 (1.13-2.13)	.007		1.67 (1.28-2.18)	<.001
**Attending health education courses**								
	No	Ref		Ref			Ref	
	Yes	0.76 (0.27-2.14)	.60	2.14 (1.33-3.45)	.002		1.73 (1.09-2.74)	.02

^a^VND: Vietnamese Dong. 1 million VND=US $44.50.

After adjusting for potential confounding variables using multivariable logistic regression for all 993 participants, we found that those obtaining health information by watching television or using the Internet were 1.7 times more likely to have an inpatient hospitalization (television: adjusted OR [AOR] 1.69, 95%CI 1.22-2.34; Internet: AOR 1.62, 95% CI 1.16-2.27) and visit a doctor (television: AOR 1.69, 95% CI 1.30-2.19; Internet: AOR 1.64, 95% CI 1.23-2.19), and those who attended health education courses were 2.1 times more likely to have inpatient hospitalizations (AOR 2.09, 95% CI 1.29-3.40) (see Table 3).

**Table 3:** Multivariable association between health care utilization and respondents’ characteristics plus the availability of health information sources: adjusted odds ratios.

Model			Emergency visit		Hospitalization		Doctor visit	
			AOR^a^ (95% CI)	*P*	AOR (95% CI)	*P*	AOR (95% CI)	*P*
**Model 1**								
	**Self-reported health status**							
		Good	Ref		Ref		Ref	
		Moderate	2.00 (0.95-4.21)	.07	1.09 (0.76-1.56)	.63	1.27 (0.95-1.69)	.10
		Poor	5.75 (2.31-14.28)	<.001	2.08 (1.17-3.70)	.01	3.00 (1.72-5.23)	<.001
	**Health insurance**							
		No	Ref		Ref		Ref	
		Yes	1.19 (0.54-2.62)	.66	1.70 (1.01-2.86)	.046	2.79 (1.89-4.13)	<.001
	**Monthly income (VND)**^b^							
		<3 million	Ref		Ref		Ref	
		3-7 million	0.77 (0.43-1.38)	.38	0.93 (0.67-1.31)	.69	1.08 (0.82-1.42)	.58
		>7 million	0.42 (0.10-1.81)	.24	1.15 (0.63-2.07)	.65	2.13 (1.22-3.74)	.008
	**Watching medical-related TV series**							
		No	Ref		Ref		Ref	
		Yes	1.24 (0.72-2.13)	.45	1.69 (1.22-2.34)	.002	1.69 (1.30-2.19)	<.001
**Model 2**								
	**Self-reported health status**							
		Good	Ref		Ref		Ref	
		Moderate	2.07 (0.98-4.38)	.06	1.18 (0.83-1.69)	.36	1.38 (1.03-1.84)	.03
		Poor	6.06 (2.39-15.35)	<.001	2.39 (1.33-4.27)	.003	3.38 (1.93-5.92)	<.001
	**Health insurance**							
		No	Ref		Ref		Ref	
		Yes	1.24 (0.57-2.70)	.60	1.84 (1.10-3.08)	.02	3.00 (2.04-4.43)	<.001
	**Monthly income (VND)**							
		<3 million	Ref		Ref		Ref	
		3-7 million	0.77 (0.43-1.38)	.39	0.94 (0.67-1.31)	.70	1.08 (0.83-1.42)	.57
		>7 million	0.41 (0.09-1.80)	.24	1.12 (0.61-2.03)	.72	2.09 (1.19-3.67)	.01
	**Getting medical-related information from the Internet**							
		No	Ref		Ref		Ref	
		Yes	1.21 (0.66-2.20)	.54	1.62 (1.16-2.27)	.005	1.64 (1.23-2.19)	.001
**Model 3**								
	**Self-reported health status**							
		Good	Ref		Ref		Ref	
		Moderate	2.01 (0.95-4.24)	.07	1.11 (0.78-1.59)	.01	1.29 (0.97-1.72)	<.001
		Poor	5.73 (2.30-14.23)	<.001	2.07 (1.17-3.67)	.56	2.93 (1.69-5.09)	.08
	**Health insurance**							
		No	Ref		Ref		Ref	
		Yes	1.26 (0.58-2.76)	.56	1.83 (1.09-3.06)	.02	3.04 (2.07-4.48)	<.001
	**Monthly income (VND)**							
		<3 million	Ref		Ref		Ref	
		3-7 million	0.79 (0.44-1.42)	.44	0.92 (0.65-1.29)	.62	1.08 (0.83-1.42)	.56
		>7 million	0.44 (0.10-1.90)	.27	1.30 (0.73-2.35)	.38	2.43 (1.40-4.22)	.002
	**Attending health education courses**							
		No	Ref		Ref		Ref	
		Yes	0.79 (0.28-2.28)	.67	2.09 (1.29-3.40)	.003	1.57 (0.98-2.50)	.06

^a^Adjusted for self-reported health status, presence of health insurance, and monthly income.

^b^VND: Vietnamese Dong. 1 million VND=US $44.50.

## Discussion

### Principal Finding

This study revealed the effect of a wider availability of mass media sources (television, Internet) for health information on health care utilization. We found that television was still a commonly reported source of health information and that the Internet was rapidly becoming a popular source of health information among Vietnamese adults. After controlling for health status, the presence of health insurance, and monthly income using a multivariable logistic regression analysis, the use of mass media sources for health information was found to be associated with higher health care utilization, especially the odds of doctor visits.

We realized that people continue to prefer traditional media (eg, television) to obtain health information, although an increasing number of people also use the Internet for health purposes [7,13]. Generally, radio is also identified as a traditional medium. However, it was observed that Vietnamese utilization of radio for news was down marginally from 27.6% in 2012/2013 to 24.8% in 2015 and that AM radio was used weekly by 3.8% of Vietnamese and shortwave by 1% [14,15]. This traditional medium is not commonly used to obtain health information. Thus, this study only focused on the impact of television as a traditional medium on health care utilization. The majority of participants in this study used television (50.9%) as a health information source, although the Internet was also used as a source of health information (32.9%); health education courses were also available. Comparing online programs related to health information, which appear to adapt to users’ general needs, and traditional health education channels is important. Similar findings regarding the prevalence of users watching medical-related television programs have been observed in Hong Kong and Japan [7,16]. On the other hand, we found that the prevalence of using the Internet for health information in Vietnam was similar or higher than that in other Asian studies in Hong Kong (30.6% in 2009, 38.2% in 2010, and 38.0% in 2012) [16] and Japan (24% in 2007) [7] and lower than that in the United States (58% in 2005, 71% in 2015) [2,17,18], Europe (52% in 2007) [19], and Poland (66.7% in 2012) [20]. Technology advancement in recent times has widely contributed in the improvement of health information dissemination not only through delivery of key health messages, but also enabling interactions among users through the different kinds of mass media.

Since the economic reform of Vietnam (known as *Doi Moi*) in 1986, the country has undergone rapid growth and an improvement in living standards in general, with more equal access to health care services in particular. Health care utilization represents a complex picture in the context of the rapidly developing economic situation and globalization in Vietnam. Self-treatment is still common in Vietnam, accounting for 40% to 60% of all treatments [21,22], although the Ministry of Health has reported overcrowding in hospitals, which have been operating at nearly 150% of their capacity. The size of the health care workforce in Vietnam is relatively small compared with other countries, based on the World Health Organization’s standard estimated number of health workers needed to provide adequate primary care coverage, which is at least 2.5 medical staff per 1000 population [23]. The number of physicians per 1000 population in Vietnam was 1.19 in 2013, whereas it was 3.03 in Japan in 2012 and 3.27 in Australia in 2011 [24]. Despite the mobilization of the private sector resources for health services and improvements in the efficiency of health systems in recent years [25], there remains a lack of health care services, insufficient public health education, no control over pharmaceutical promotions, and a lack of efficient drug policies and regulations. Hence, both the underuse of health care services and overcrowding in central hospitals are simultaneously present in Vietnam.

In this context, improving the utilization of health care services among residents depends on not only the availability and quality of services, but also the preferences of users, which are based on their experiences, health status, socioeconomic characteristics, and the availability of health information. The utilization of health care services appears to differ according to the self-reported health status, the presence of health insurance, household economic situation, similar to observations in previous studies conducted in Vietnam and other countries [21,26-28]. In this study, differences in health care utilization, including emergency department visits, hospitalizations, and doctor visits, were observed among self-reported health status subgroups and health insurance users; moreover, the group with higher monthly income of more than 7 million VND had a higher frequency of doctor visits than the poorer groups, but significant differences in the frequency of emergency department and inpatient visits were not observed.

The demand for health care expands from seeking health information to utilizing the health care facilities. We were impressed by the effect of a wider availability of mass media sources for health information on the frequency of hospitalization and doctor visits even after adjustments for the self-reported health status, the presence of health insurance, and monthly income. The use of mass media sources (eg, television) and the Internet to obtain health information was associated with higher health care utilization among the general public. Obtaining information through these mass media sources to increase health care knowledge, attitudes, and suitable health care use is expected to be important for health care promotion [29,30], particularly for reducing self-treatment in Vietnam, although a gap between the quality and quantity of health services and the demand for health care continues to exist.

Mass media sources could potentially play a role in raising awareness about health care services and in shaping the perceptions of health care and related decision-making [30,31]. Similar findings in previous reports provide strong evidence to support this point. In 2008, Lee et al [1] reported that the increasing use of the Internet was correlated with an increase in outpatient clinic visits after controlling for age and sex in a data analysis. Other authors have also elucidated a significant association between use of health information technology and health services use [29,31,32]. First, we recognized that the availability of health information in mass media sources has triggered the health care interest of the general population [33]. People needed more help from health care professionals with interpreting and understanding the health information they obtained. Medical knowledge obtained from the Internet, in particular, might not be as reliable as information published in academic journals [34] or is less likely to be accredited by medical professors. With a multitude of messages available through a wide variety of mass media sources, inconsistencies can lead to confusion among audiences [35]. As a result, people who use mass media sources might be more likely to visit doctors, especially as outpatients, to clarify and interpret health information. Second, other studies have suggested that people seeking health information through mass media sources are more likely to evaluate themselves as having poor health and may have a greater tendency to seek a doctor’s advice regarding their health status [6,31]. Therefore, physicians may need to spend additional time discussing medical topics, interpreting symptoms or health information, and assuring patients.

In addition, based on the multivariable logistic regression analysis, there was also significant association between use of available health information through health education courses and inpatient hospitalization (*P*=.003). The availability of health information through community health workers is a notable strategy to improve health care utilization. To further support health education in the community, the Ministry of Health through Circular 07 declared that every health worker, regardless of where they work, should receive at least 24 hours of updated professional training [36,37] to improve the quality of health worker and skill to transmit health information to the public. However, there are challenges to focus on this resource to expand the interest in health information compared with more widely available mass media sources.

### Limitations

Although the results of this study extend what is already known and provide new data in the Vietnamese context by controlling for confounding variables, there are several study limitations. First, this cross-sectional study used a structured questionnaire requiring the participants to recall the frequency of use of mass media sources to obtain health information and health care utilization and thus cannot speculate on the order of causality unlike a cohort study design and a recall bias might have affected the results. The assessment would have been more reliable if self-reported information and clinical examinations or health record profiles had been integrated. To ensure the quality of data, we provided the interviewers with rigorous training on interviewing techniques and continually monitored the data quality during the fieldwork process. Second, the data were only collected in one place (city of Hue) and might not be representative of the whole country. Further study involving a nationally representative cohort is needed to provide comprehensive evidence. Finally, factors concerning the quality of health information obtained from mass media sources and used by participants were not examined in this study.

### Conclusions

Notwithstanding the previously mentioned limitations, this study found that a wider availability of mass media sources for health information was associated with higher health care utilization. The diverse nature of health information available through mass media sources might play an important role in the frequency of clinic visits. Despite the gap between demand for health care and the quality of health care services, expansion of interest in health care is likely to be useful for further promotion of health in developing countries.

## Abbreviations

AOR: adjusted odds ratio
